# Supplementary material for: Beclin‐1‐mediated activation of autophagy improves proximal and distal urea cycle disorders
Source: EMBO Mol Med. 2020 Dec 28;13(2):e13158. doi: 10.15252/emmm.202013158 (PMC7863400; doi:10.15252/emmm.202013158)
Supplement: Supplementary file 1 — Appendix [file EMMM-13-e13158-s001.pdf]

# APPENDIX

*Beclin-1-mediated activation of autophagy improves proximal and distal urea cycle disorders.*

Leandro R. Soria (*a*), Sonam Gurung (*b*), Giulia De Sabbata (*c*), Dany P. Perocheau (*b*), Angela De Angelis (*a*), Gemma Bruno (*a*), Elena Polishchuk (*a*), Debora Paris (*d*), Paola Cuomo (*d*), Andrea Motta (*d*), Michael Orford (*b*), Youssef Khalil *(b)*, Simon Eaton (*b*), Philippa B. Mills *(b)*, Simon N. Waddington (*b, e*), Carmine Settembre (*a*), Andrés F. Muro (*c*), Julien Baruteau *(b, f)*, and Nicola Brunetti-Pierri (*a, g*).

***(a)*** Telethon Institute of Genetics and Medicine, Pozzuoli, Italy; ***(b)*** UCL Great Ormond Street Institute of Child Health, London, UK; ***(c)*** International Centre for Genetic Engineering and Biotechnology, Trieste, Italy; ***(d)*** Institute of Biomolecular Chemistry, National Research Council, Pozzuoli, Italy; ***(e)*** Wits/SAMRC Antiviral Gene Therapy Research Unit, Faculty of Health Sciences, University of the Witwatersrand, Johannesburg, South Africa; ***(f)*** Metabolic Medicine Department, Great Ormond Street Hospital for Children NHS Foundation Trust, London, UK; ***(g)*** Department of Translational Medicine, Federico II University, Naples, Italy.

**Content:**
Appendix Figure S1
Appendix Table S1

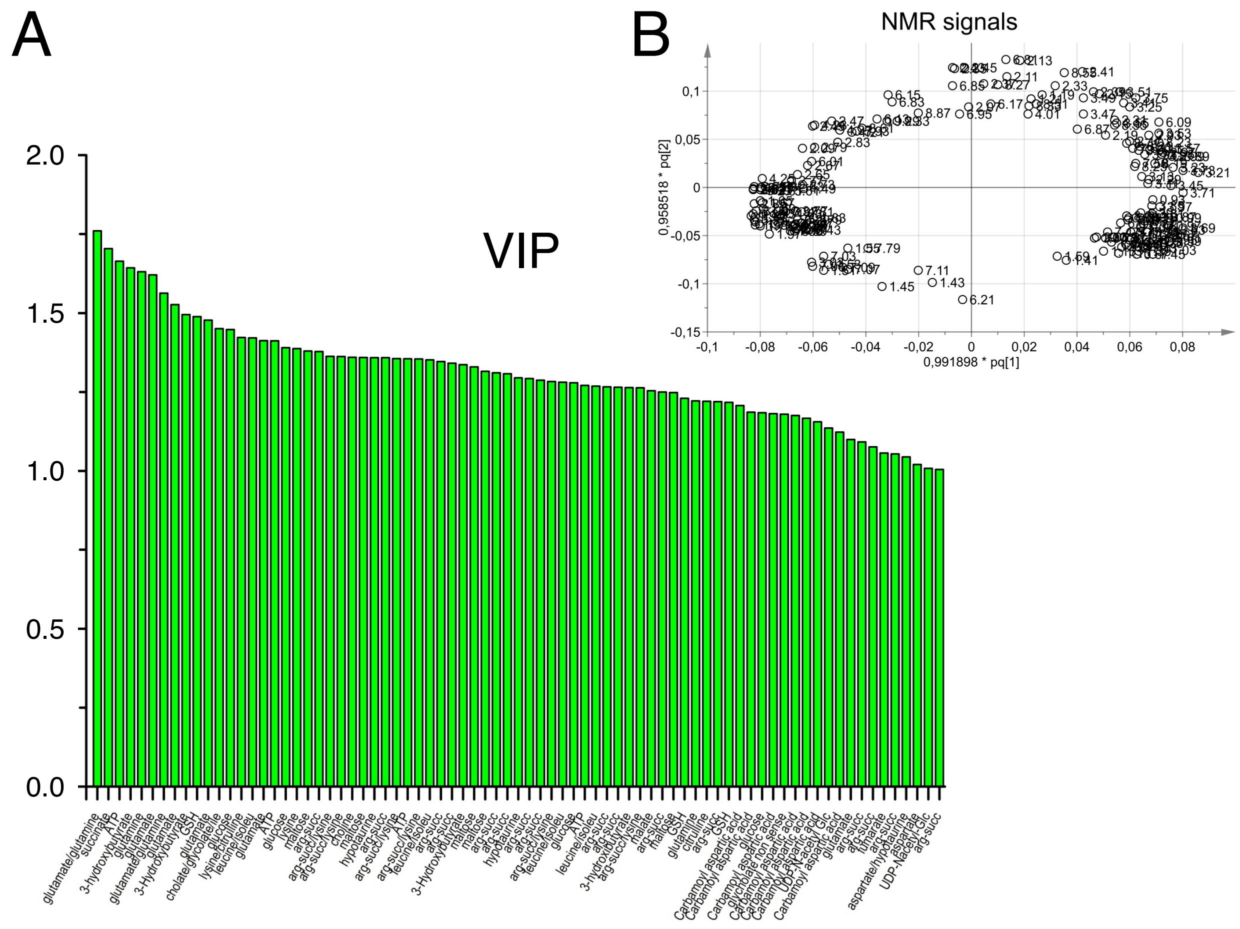


**Appendix Figure S1. Liver metabolome extended data.** (**A**) Variable importance of projection (VIP) score plot, and loadings plot (**B**) of the Orthogonal Projection to Latent Structure-Discriminant Analysis (OPLS-DA) model showed in **Fig. 2G**. For the analysis, statistically significant metabolites responsible for the separation between groups were selected from those indicated on the x-axis in **(A)** by using a combination of VIP value >1 and correlation loadings values p(corr) > 0.7, as previously reported (Soria LR, Allegri G, Melck D, Pastore N, Annunziata P, Paris D, Polishchuk E, Nusco E, Thony B, Motta A et al (2018) Enhancement of hepatic autophagy increases ureagenesis and protects against hyperammonemia. *Proc Natl Acad Sci U S A* 115**:** 391-396).

**Appendix Table S1.** Summary of significant P values for Main and EV figures.

| | |
|---|---|
| **Fig. 1A** | p=0.00649424 |
| **Fig. 1B** | p=0.019 |
| **Fig. 1D** (LC3II) | p<0.0001 |
| **Fig. 1D** (NBR1) | p=0.003561333 |
| **Fig. 1D** (p62) | p=0.02190909 |
| **Fig. 1E** | p=0.0009 |
| **Fig. 1F** (WT vs. OTC unt) | p=0.0192 |
| **Fig. 1F** (OTC unt vs. OTC + scavenger + L-Arg) | p=0.0022 |
| **Fig. 1F** (OTC unt vs. OTC + TB-1 + scavenger + L-Arg) | p<0.0001 |
| **Fig. 1F** (OTC + TB-1 vs. OTC + TB-1 + scavenger + L-Arg) | p=0.0016 |
| | |
| **Fig. 2A** | p=0.028 |
| **Fig. 2B** | p=0.033636344 |
| **Fig. 2D** (LC3II) | p=0.008968393 |
| **Fig. 2D** (NBR1) | p=0.023319683 |
| **Fig. 2D** (p62) | p=0.04294431 |
| **Fig. 2E** (WT vs. AslNeo/Neo) | p=0.0004 |
| **Fig. 2E** (WT vs. AslNeo/Neo + TB-1) | p=0.0222 |
| **Fig. 2E** (AslNeo/Neo vs. AslNeo/Neo + TB-1) | p=0.0452 |
| **Fig. 2F** | p=0.04610307 |
| | |
| **Fig. 3B** (WT vs. AslNeo/Neo) | p<0.0001 |
| **Fig. 3B** (WT vs. AslNeo/Neo + TB-1) | p=0.0081 |
| **Fig. 3B** (AslNeo/Neo vs. AslNeo/Neo + TB-1) | p=0.012 |
| **Fig. 3C** (WT vs. AslNeo/Neo) | p=0.0003 |
| **Fig. 3C** (WT vs. AslNeo/Neo + TB-1) | p=0.017 |
| **Fig. 3C** (AslNeo/Neo vs. AslNeo/Neo + TB-1) | p=0.0447 |
| **Fig. 3D** (WT vs. AslNeo/Neo) | p=0.0415641 |
| **Fig. 3D** (WT vs. AslNeo/Neo + TB-1) | p=0.0164057 |
| | |
| **Fig. EV1A** | p=0.0163 |
| | |
| **Fig. EV2D** (OTC + TB-1 D4 vs. OTC + scavenger + L-Arg D4) | p=0.0092 |
| **Fig. EV2D** (OTC + scavenger + L-Arg D0 vs. OTC + scavenger + L-Arg D4) | p=0.0006 |
| **Fig. EV2D** (OTC + scavenger + L-Arg D4 vs. OTC + TB-1 + scavenger + L-Arg D4) | p=0.0049 |
| | |
| **Fig. EV3B** | p=0.0370 |
| **Fig. EV3C** (WT vs. AslNeo/Neo) | p=0.0010053 |
| **Fig. EV3C** (WT vs. AslNeo/Neo + TB-1) | p=0.0013328 |
| **Fig. EV3C** (WT +TB-1 vs. AslNeo/Neo) | p=0.0015198 |
| **Fig. EV3C** (WT +TB-1 vs. AslNeo/Neo + TB-1) | p=0.0032734 |
| | |
| **Fig. EV4** (argininosuccinate) | WT vs. Asl p=0.0010053; Asl vs. Asl TB-1 p=0.0010053 |
| **Fig. EV4** (citrulline) | WT vs. Asl p=0.0149443; Asl vs. Asl TB-1 p=0.0189921 |
| **Fig. EV4** (aspartate) | WT vs. Asl p=0.0014606; Asl vs. Asl TB-1 p=0.0234101 |
| **Fig. EV4** (fumarate) | WT vs. Asl p=0.0046971 |
| **Fig. EV4** (lysine) | WT vs. Asl p=0.0118262; Asl vs. Asl TB-1 p=0.0031376 |
| **Fig. EV4** (ATP) | - |
| **Fig. EV4** (glucose) | WT vs. Asl p=0.0011964; Asl vs. Asl TB-1 p=0.007925 |
| **Fig. EV4** (succinate) | WT vs. Asl +TB1 p=0.0403553; Asl vs. Asl TB-1 p=0.0010053 |
| **Fig. EV4** (glutamate) | WT vs. Asl +TB1 p=0.0173686; Asl vs. Asl TB-1 p=0.0476236 |
| **Fig. EV4** (glutamine) | WT vs. Asl +TB1 p=0.0081175; Asl vs. Asl TB-1 p=0.0166672 |
| **Fig. EV4** (hypotaurine) | WT vs. Asl p=0.0010053; Asl vs. Asl TB-1 p=0.005165 |
| **Fig. EV4** (GSH) | Asl vs. Asl TB-1 p=0.005165 |
| **Fig. EV4** (leucine/isoleucine) | WT vs. Asl p=0.0015791; WT vs. Asl TB-1 p=0.0105148 |
| **Fig. EV4** (3-hydroxybutyrate) | - |
| **Fig. EV4** (UDP-N-acetyl-Glc) | WT vs. Asl p=0.0010053; Asl vs. Asl TB-1 p=0.0010053 |
| **Fig. EV4** (maltose) | WT vs. Asl p=0.0010053; WT vs. Asl TB-1 p=0.0156078 |
| | |
| **Fig. EV5B** (WT vs. AslNeo/Neo) | p=0.0003 |
| **Fig. EV5B** (WT vs. AslNeo/Neo + TB-1) | p=0.0023 |
| **Fig. EV5C** (WT vs. AslNeo/Neo) | p=0.0412 |
