# Supplementary material for: Beclin‐1‐mediated activation of autophagy improves proximal and distal urea cycle disorders
Source: EMBO Mol Med. 2020 Dec 28;13(2):e13158. doi: 10.15252/emmm.202013158 (PMC7863400; doi:10.15252/emmm.202013158)
Supplement: Supplementary file 2 — Expanded View Figures PDF [file EMMM-13-e13158-s002.pdf]

Expanded View Figures

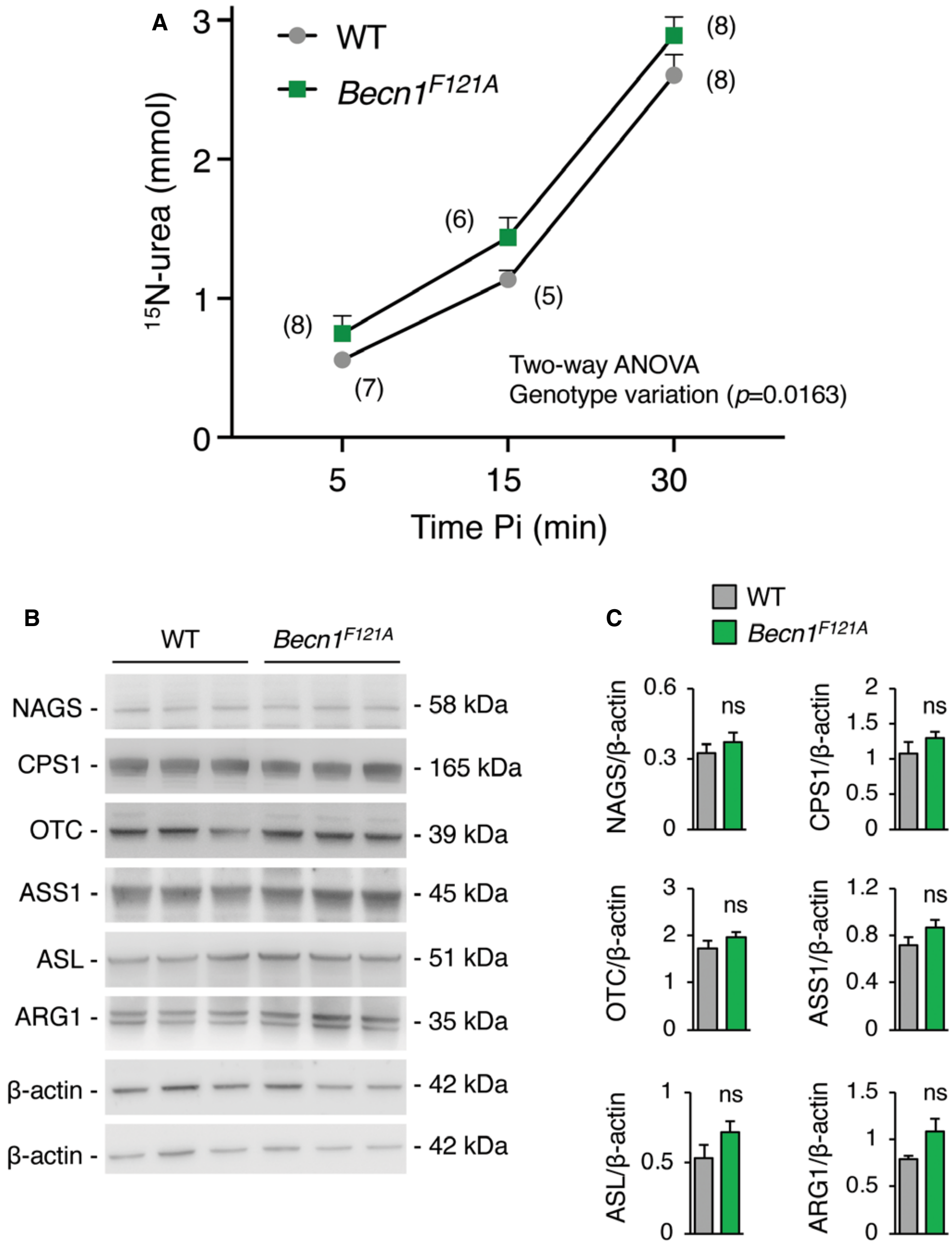

Figure EV1. Ureagenesis in *Becn1*^{F121A} mice.

A ^{15}N-labelled urea in blood at 5, 15 and 30 min after i.p. injection of $^{15}NH_4Cl$ tracer (10 mmol/kg) in 8–13-week-old wild-type (WT) and *Becn1*^{F121A} mice. The number between parentheses indicates the number of mice per time-point. Two-way ANOVA analysis showed a significant difference between the two mice genotypes.

B Western blot analyses of urea cycle enzymes (NAGS, CPS1, OTC, ASS1, ASL, and ARG1) in livers of WT and *Becn1*^{F121A} mice. β-actin was used as loading control: upper β-actin blot for CPS1, OTC and ASS1; lower β-actin blot for NAGS, ASL and ARG1.

C Densitometric quantifications (n = 4 mice/group) (Unpaired t-test).

Data information: All values are shown as averages ± SEM. ns: not statistically significant difference.
Source data are available online for this figure.

© 2021 The Authors

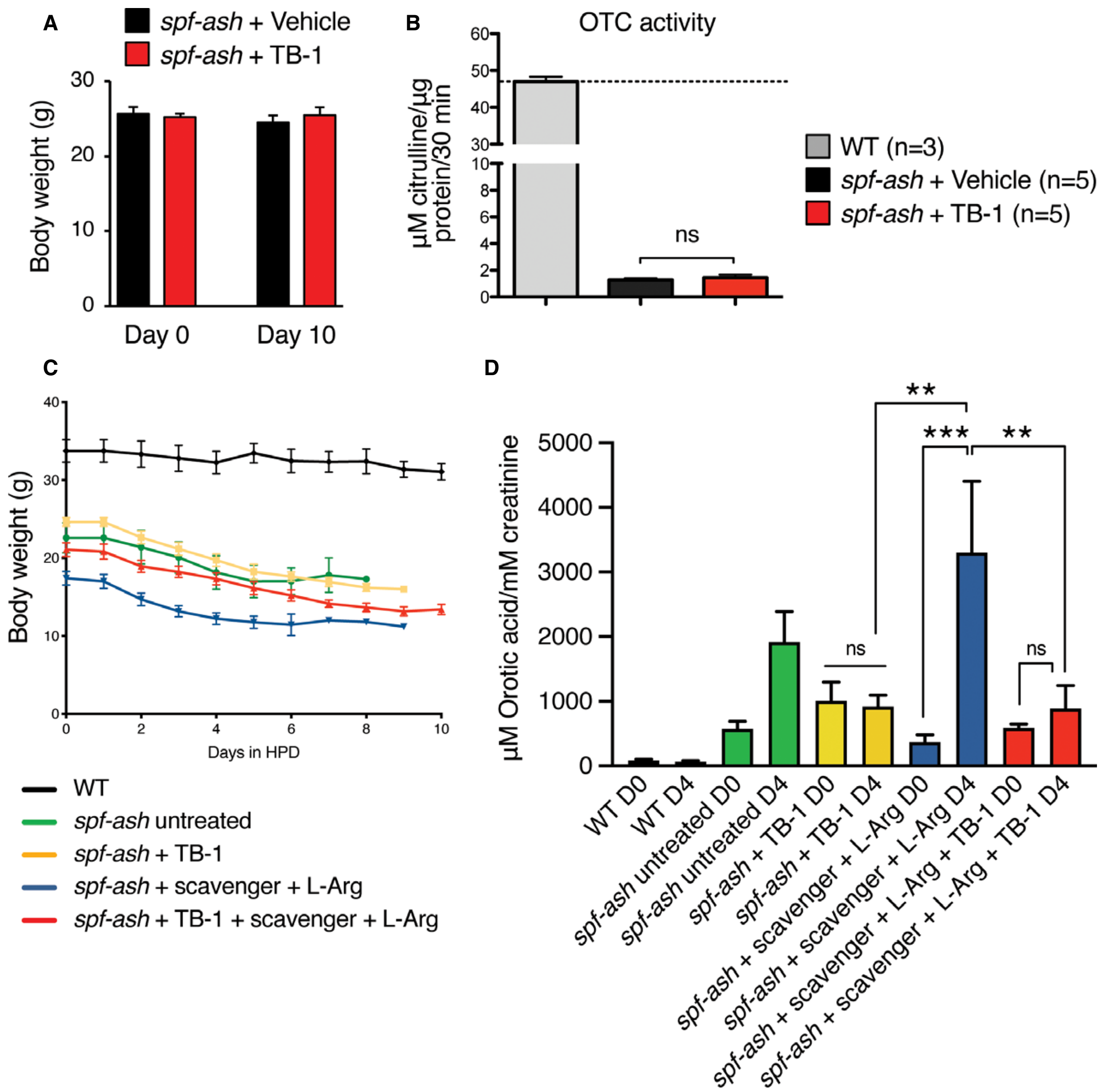

Figure EV2. TB-1 treatment in *spf-ash* mice.

A Body weights of *spf-ash* mice at baseline and up to day 10 of treatment with Tat-Beclin-1 (TB-1) (related to Fig 1B) (n = 5 mice/group).

B Liver ornithine transcarbamylase (OTC) catalytic activity in *spf-ash* mice injected with TB-1 or vehicle (n = 5 mice/group) compared to wild-type (WT) levels (n = 3) (Unpaired t-test).

C Body weights of WT and *spf-ash* mice fed with a high protein diet (HPD) (related to Fig 1E) (n = 4–6 mice/group).

D Urinary orotic acid levels at baseline (D0) and after 4 days of HPD (D4) (related to Fig 1E) ($n \geq 4$ mice/group); ***$P < 0.001$, **$P < 0.01$ (One-way ANOVA).

Data information: All values are shown as averages ± SEM. ns: not statistically significant difference. Exact P values are reported in Appendix Table S1.
Source data are available online for this figure.

 © 2021 The Authors

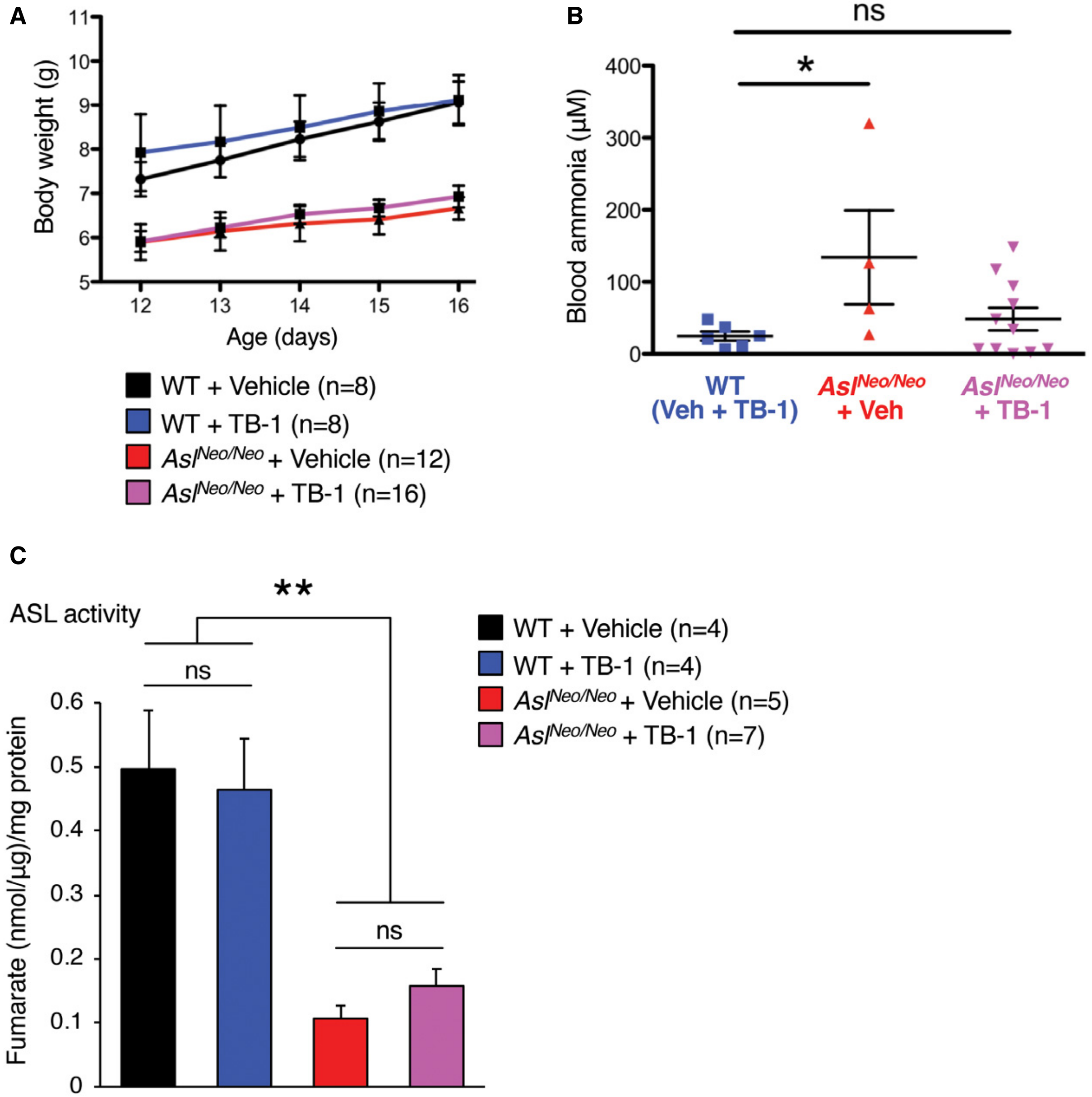

Figure EV3. Short-term treatment of TB-1 in ASL-deficient mice.

A Body weights of wild-type (WT) and ASL-deficient (*Asl*$^{Neo/Neo}$) mice injected with Tat-Beclin-1 (TB-1) or vehicle (n = 8–16 mice/group).

B Blood ammonia in WT and *Asl*$^{Neo/Neo}$ mice injected with TB-1 or vehicle (n = 4–11 mice/group). *$P < 0.05$ (One-way ANOVA with Dunnett's post-test compared to WT). ns: not statistically significant difference.

C Liver arginosuccinate lyase (ASL) catalytic activity in WT and *Asl*$^{Neo/Neo}$ mice injected with TB-1 or vehicle (n = 4–7 mice/group). ASL residual activity is unaffected in TB-1-treated mice. **$P < 0.01$ (One-way ANOVA).

Data information: All values are shown as averages ± SEM. Exact P values are reported in Appendix Table S1.
Source data are available online for this figure.

© 2021 The Authors

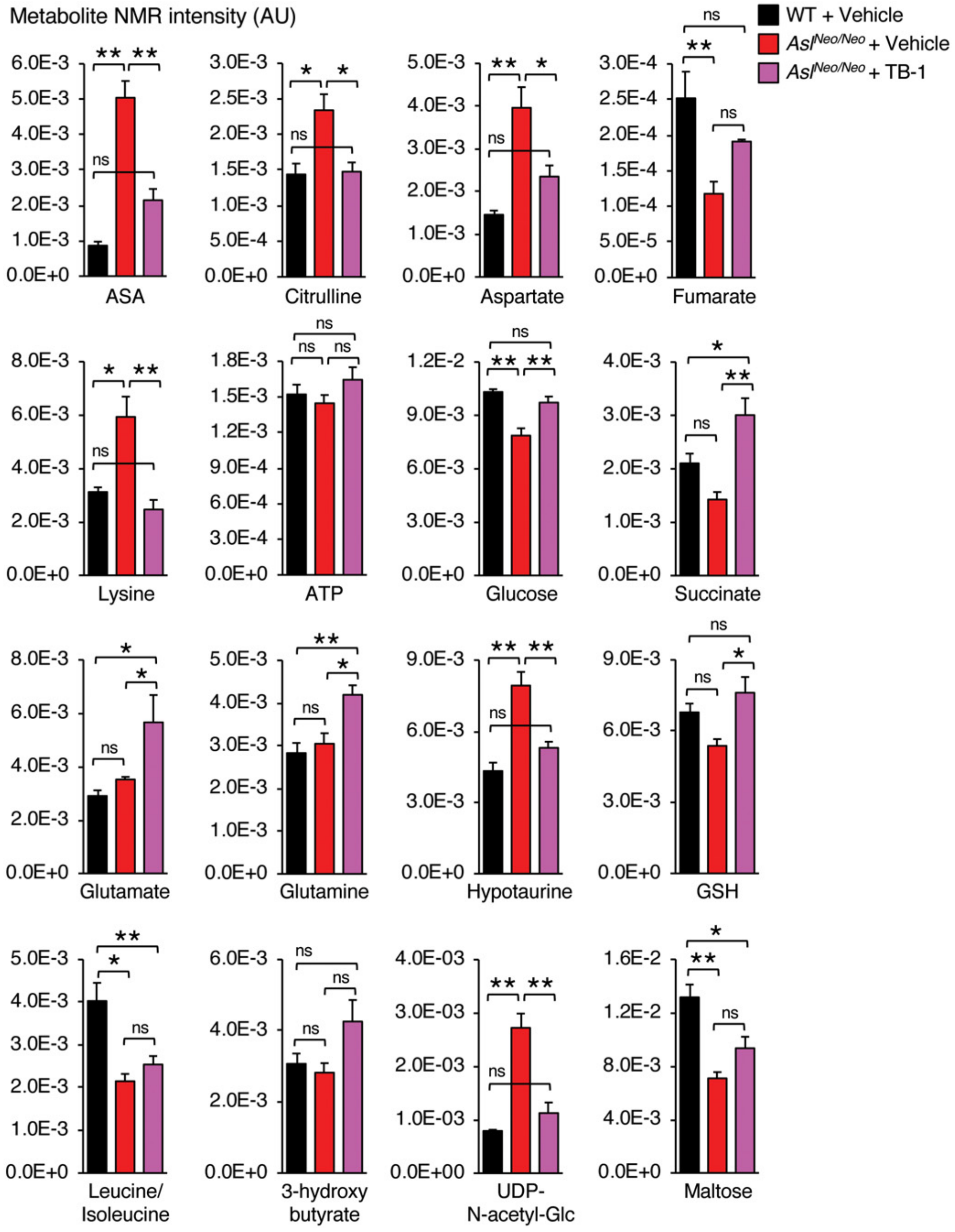

Figure EV4. Enhancement of liver autophagy corrects the metabolic defects of *Asl$^{Neo/Neo}$* mice.

Liver metabolites acquired and quantified through ^{1}H-NMR analysis with statistically significant differences between control wild-type (WT) compared to ASL-deficient (*Asl$^{Neo/Neo}$*) mice treated with Tat-Beclin-1 (TB-1) or vehicle ($n \geq 4$ mice/group). See also Appendix Fig S1. The values are shown as averages ± SEM. $^{**}P < 0.01$, $^{*}P < 0.05$ (One-way ANOVA). ASA: argininosuccinic acid; ns: not statistically significant difference. Exact *P* values are reported in Appendix Table S1.

Source data are available online for this figure.

 © 2021 The Authors

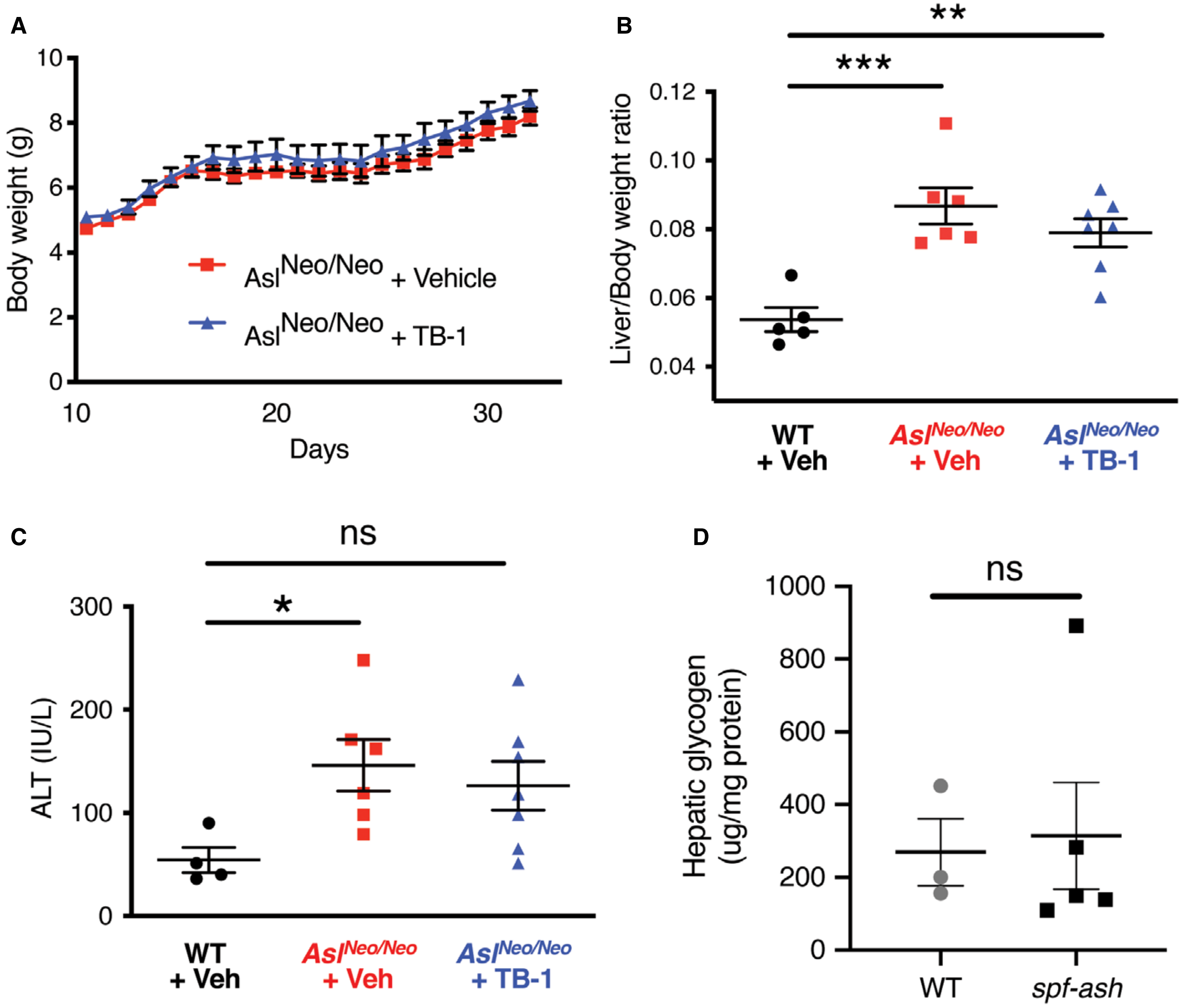

Figure EV5. Long-term treatment of TB-1 in ASL-deficient mice.

A Body weights (related to Fig 3A) of ASL-deficient (*Asl*$^{Neo/Neo}$) mice treated with Tat-Beclin-1 (TB-1, n = 12) or vehicle (n = 11).

B Liver/body weight ratio in wild-type (WT) and *Asl*$^{Neo/Neo}$ mice injected with TB-1 or vehicle (n = 4–7 mice/group). ***$P < 0.001$, **$P < 0.01$ (One-way ANOVA with Dunnett's post-test compared to WT).

C Circulating levels of alanine aminotransferase (ALT) in WT and *Asl*$^{Neo/Neo}$ mice injected with TB-1 or vehicle (n = 4–7 mice/group). *$P < 0.05$, (One-way ANOVA with Dunnett's post-test compared to WT).

D Liver content of glycogen in *spf-ash* mice (n = 5) compared to wild-type (WT) (n = 3) (Unpaired t-test).

Data information: The values are shown as averages ± SEM. ns: not statistically significant difference. Exact P values are reported in Appendix Table S1.
Source data are available online for this figure.
