# Supplementary material for: Beclin‐1‐mediated activation of autophagy improves proximal and distal urea cycle disorders
Source: EMBO Mol Med. 2020 Dec 28;13(2):e13158. doi: 10.15252/emmm.202013158 (PMC7863400; doi:10.15252/emmm.202013158)
Supplement: Supplementary file 3 — Source Data for Expanded View [file EMMM-13-e13158-s007.zip › SD_EV5.pdf]

## EV 5A

| AslNeo/Neo + Veh | | | | | | | | | | | AslNeo/Neo + TB-1 | | | | | | | | | | | |
|---|---|---|---|---|---|---|---|---|---|---|---|---|---|---|---|---|---|---|---|---|---|---|
| 4.9 | 4.7 | 4.8 | 4.4 | 5.1 | 5.1 | 4.3 | 4.88 | | | 4.7 | 4.6 | 4.7 | 4.6 | 4.6 | 4.67 | 5.14 | 5.68 | 4.9 | 4.77 | 5.27 | 5.9 | 5.8 |
| 5 | 4.9 | 5 | 4.5 | 5 | 5.2 | 4.85 | 5.1 | 5.38 | 5.55 | 4.8 | 4.6 | 4.8 | 4.6 | 4.2 | 5.1 | 5.12 | 5.7 | 5.1 | 4.96 | 5.25 | 6.02 | 5.85 |
| 5.67 | 5.08 | 4.94 | | | 5.4 | 4.76 | 5.06 | 5.64 | 5.63 | 5.04 | 4.69 | 5.04 | 4.69 | 4.31 | 5 | 5.3 | 5.81 | 5.71 | 4.85 | 5.9 | 6.09 | 6.75 |
| 6.03 | 5.6 | 5.8 | | | 6 | | 4.85 | 5.8 | 5.87 | 5.59 | 5.17 | 5.59 | 5.17 | 4.95 | 5.64 | 5.6 | 6.37 | 5.9 | | 6.2 | 6.9 | 7.38 |
| 6.26 | 5.95 | 6.27 | | | 6.81 | | | 6.38 | 6.8 | 5.76 | 5.28 | 5.76 | 5.28 | 5.15 | 6.23 | 5.56 | 6.61 | 6.31 | | 6.94 | 7.62 | 7.87 |
| 6.75 | 6.09 | 6.11 | | | 6.98 | | | 7.07 | 7.16 | 6.4 | 5.63 | 6.4 | 5.63 | 5.4 | 6.51 | 5.56 | 6.75 | 6.8 | | 7.08 | 7.83 | 8.51 |
| 6.45 | 6 | 6.1 | | | 7.14 | | | 6.99 | 7.44 | 6.26 | 5.55 | 6.26 | 5.55 | 5.58 | 7.08 | | 6.99 | 6.6 | | 7.41 | 7.9 | 9 |
| 6.33 | 6.07 | 6 | | | 7.02 | | | 6.77 | 7.21 | 6.2 | 5.32 | 6.2 | 5.32 | 5.43 | 6.49 | | 6.95 | 6.96 | | 7.37 | 7.98 | 9.12 |
| 6.5 | 6.4 | 6.4 | | | 6.95 | | | 6.65 | 7.1 | 6.22 | 5.4 | 6.22 | 5.4 | 5.45 | 6.53 | | 6.94 | 6.88 | | 7.3 | 8.26 | 9.7 |
| 6.6 | 6.5 | 6.4 | | | 6.9 | | | 6.65 | 7.14 | 6.2 | 5.5 | 6.2 | 5.5 | 5.5 | 6.5 | | 7.1 | 6.8 | | 7.1 | 8.71 | 9.82 |
| 6.7 | 6.4 | 6.3 | | | 6.92 | | | 6.83 | 7.39 | 6.2 | 5.5 | 6.2 | 5.5 | 5.6 | 6.3 | | 6.8 | 6.68 | | 6.99 | 8.2 | 9.6 |
| 6.43 | 6.29 | 6.12 | | | 7 | | | 6.93 | 7.41 | 6.06 | 5.32 | 6.06 | 5.32 | 5.38 | 6.56 | | 6.32 | 6.45 | | 7.13 | 8.68 | 9.64 |
| 6.46 | 6.23 | 6.12 | | | 7.27 | | | 7.12 | 7.55 | 6.14 | 5.28 | 6.14 | 5.28 | 5.35 | 6.77 | | 6.74 | 6.75 | | 6.96 | 8.47 | 9.56 |
| 5.91 | 5.66 | 5.92 | | | 7.48 | | | 7.46 | 7.48 | 5.63 | 6.05 | 5.63 | 6.05 | 4.91 | 6.96 | | 6.43 | 6.4 | | 6.7 | 8.68 | 9.65 |
| 6.67 | 6.62 | 6.45 | | | 7.92 | | | 7.2 | 7.36 | 6.17 | 5.35 | 6.17 | 5.35 | | 7.34 | | 6.7 | 6.51 | | 6.89 | 8.52 | 9.58 |
| 6.7 | 6.55 | 6.39 | | | 8.35 | | | 7.03 | 7.2 | 6.22 | 5.87 | 6.22 | 5.87 | | 7.8 | | 6.94 | 6.71 | | 6.94 | 8.22 | 9.17 |
| 7 | 6.9 | 6.6 | | | 8.54 | | | 7 | 7.22 | 6.3 | 5.5 | 6.3 | 5.5 | | 8.2 | | 7.88 | 6.7 | | 6.99 | 8.62 | 9.8 |
| 7.66 | 7.28 | 6.99 | | | 8.6 | | | 6.92 | 7.23 | 6.47 | 6.47 | 6.47 | 6.47 | | 8.1 | | 8.04 | 7.27 | | 7.47 | 8.2 | 9.6 |
| 8.1 | 7.61 | 7.36 | | | 9.24 | | | 7.1 | 7.32 | 6.84 | 6.2 | 6.84 | 6.2 | | 8.15 | | 8.4 | 7.74 | | 7.83 | 8.56 | 9.76 |
| 8.4 | 8.9 | 7.71 | | | 8.75 | | | 7 | 7.2 | 7.4 | 6.81 | 7.4 | 6.81 | | 8.15 | | 9.27 | 7.99 | | 8.65 | 8.5 | 9.7 |
| 8.8 | 8.54 | 8.28 | | | 8.8 | | | 6.9 | 7.2 | 7.49 | 7.07 | 7.49 | 7.07 | | 8.12 | | 9.35 | 8.33 | | 8.8 | 8.57 | 10.15 |
| 9.4 | 8.42 | 8.75 | | | 8.74 | | | 7.05 | 7.76 | 7.94 | 7.6 | 7.94 | 7.6 | | 7.97 | | 9.35 | 8.31 | | 9.13 | 8.8 | 10.33 |
| | | | | | | | | | | | | | | | | | | | | | | |
| | Body weight (g) | | | | | | | | | | | | | | | | | | | | | |

## EV 5B

| WT + Vehicle | AslNeo/Neo + Vehicle | AslNeo/Neo + TB-1 |
|---|---|---|
| 0.05096012 | 0.07771429 | 0.08026316 |
| 0.05 | 0.0787234 | 0.06926952 |
| 0.04642857 | 0.0760095 | 0.09159347 |
| 0.05433187 | 0.08810069 | 0.08663101 |
| 0.06666667 | 0.1108071 | 0.08066429 |
| | 0.08926261 | 0.06024097 |
| | | 0.08423586 |
| | | |
| Liver/Body weight ratio | | |

## EV 5C

| WT + Vehicle | AslNeo/Neo + Vehicle | AslNeo/Neo + TB-1 |
|---|---|---|
| 40 | 171 | 118 |
| 51 | 248 | 154 |
| 36 | 98 | 169 |
| 90 | 79 | 51 |
| | 162 | 98 |
| | 119 | 65 |
| | | 229 |
| | | |
| ALT (IU/L) | | |

## EV 5D

| WT | spf-ash |
|---|---|
| 200.23149 | 282.4442176 |
| 156.416768 | 109.3323405 |
| 451.178322 | 891.4021978 |
| | 138.7390377 |
| | 149.5194063 |
| | |
| Glycogen (ug/mg protein) | |
