# Supplementary material for: Beclin‐1‐mediated activation of autophagy improves proximal and distal urea cycle disorders
Source: EMBO Mol Med. 2020 Dec 28;13(2):e13158. doi: 10.15252/emmm.202013158 (PMC7863400; doi:10.15252/emmm.202013158)
Supplement: Supplementary file 3 — Source Data for Expanded View [file EMMM-13-e13158-s007.zip › SD_EV4.pdf]

EV 4

	ASA	Citrulline	Aspartate	Fumarate	Lysine	ATP	Glucose	Succinate	Glutamate	Glutamine	Hypotaurine	GSH	Leucine/Isoleucine	3-hydroxybutyrate	UDP-N-Acetyl Glc	Maltose
WT	0.00111671	0.00120264	0.00167061	0.00023058	0.00339361	0.00168264	0.00989354	0.00208064	0.00309449	0.0033437	0.00533617	0.00723235	0.00369367	0.00348237	0.0008101	0.01168312
WT	0.00075038	0.00143273	0.00146677	0.00035783	0.00305921	0.00144471	0.01044938	0.0025471	0.00338038	0.00305896	0.00374112	0.00753415	0.00418171	0.00334612	0.00074708	0.01174543
WT	0.00096973	0.00187438	0.00119701	0.00018236	0.00264018	0.00132924	0.01027097	0.00165226	0.00243532	0.00224864	0.00393815	0.00590562	0.00512546	0.00323559	0.0007688	0.01577467
WT	0.00062149	0.00124771	0.00149221	0.00023653	0.00337272	0.00162925	0.01063747	0.00213078	0.00269235	0.00266588	0.00428999	0.00647704	0.00311156	0.00221351	0.00084093	0.01342024
AslNeo/Neo + Veh	0.0047034	0.00209038	0.00234883	0.00018112	0.00551731	0.00123335	0.00787389	0.00118514	0.00338754	0.0020958	0.00779263	0.00552339	0.00265922	0.00267668	0.00185041	0.00748055
AslNeo/Neo + Veh	0.00496454	0.00216669	0.00493511	0.0001221	0.00452909	0.00153962	0.00845377	0.00130088	0.00363097	0.00334683	0.007207	0.00529695	0.00214965	0.00234145	0.00285239	0.0078919
AslNeo/Neo + Veh	0.00485432	0.00221408	0.00499634	0.00010403	0.00448979	0.00141755	0.00847857	0.00127547	0.00362598	0.00333146	0.00743568	0.00543941	0.00227872	0.00235774	0.00274085	0.00813105
AslNeo/Neo + Veh	0.00678438	0.00322827	0.0037772	0.00009821	0.00856001	0.0013996	0.00626833	0.00138683	0.00320523	0.0030457	0.01016973	0.00442222	0.00159193	0.00293138	0.00264375	0.00595664
AslNeo/Neo + Veh	0.00390256	0.00200587	0.00374798	0.00008284	0.00656471	0.00164378	0.00824268	0.001974	0.00373732	0.00342356	0.0070321	0.00619253	0.00206521	0.00379685	0.0035239	0.00595122
AslNeo/Neo + TB-1	0.00301192	0.00178166	0.0030411	0.00019062	0.00349484	0.00168255	0.00913286	0.00289823	0.00621911	0.00396759	0.00462124	0.00691438	0.00241088	0.00413439	0.00170774	0.00722863
AslNeo/Neo + TB-1	0.00180824	0.00130582	0.00210068	0.00019906	0.00183389	0.00163037	0.0103852	0.00315725	0.00547969	0.00432725	0.00523748	0.00811341	0.00229347	0.00392784	0.00084106	0.01009673
AslNeo/Neo + TB-1	0.00221281	0.00160308	0.00182681	0.00018783	0.00224325	0.00137723	0.01022094	0.00222795	0.00300293	0.00372268	0.0058643	0.00620167	0.00233268	0.00304438	0.00099316	0.01123612
AslNeo/Neo + TB-1	0.00154998	0.00121922	0.00242813	0.00018648	0.00227418	0.00188975	0.00913055	0.00374548	0.00793308	0.00475364	0.00548332	0.00922183	0.00312867	0.0059225	0.00096395	0.00880333
