# Supplementary material for: Beclin‐1‐mediated activation of autophagy improves proximal and distal urea cycle disorders
Source: EMBO Mol Med. 2020 Dec 28;13(2):e13158. doi: 10.15252/emmm.202013158 (PMC7863400; doi:10.15252/emmm.202013158)
Supplement: Supplementary file 3 — Source Data for Expanded View [file EMMM-13-e13158-s007.zip › SD_EV1.pdf]

EV. 1A

Min Pi	WT							
5	0.42335854	0.59702954	0.52285154	0.57841941	0.69900131	0.54986717	0.54321485	
15	1.24198902	1.25159413	1.2283975	1.03850533	0.91839893			
30	2.43361289	1.92991259	2.43069725	2.32718099	2.62476227	2.82388627	3.24549812	3.02726236
Min Pi	Becn1F121A							
5	0.54735642	0.36026288	0.4741116	0.70661984	0.85001915	0.58348779	1.48090254	0.97626488
15	1.65448012	1.5208167	0.93905983	1.21211302	1.36954946	1.93773539		
30	2.25929123	2.87404246	3.44640619	2.50470386	2.87361479	3.19537172	2.87005076	3.09002215
15N-urea (mmol)								

EV. 1B

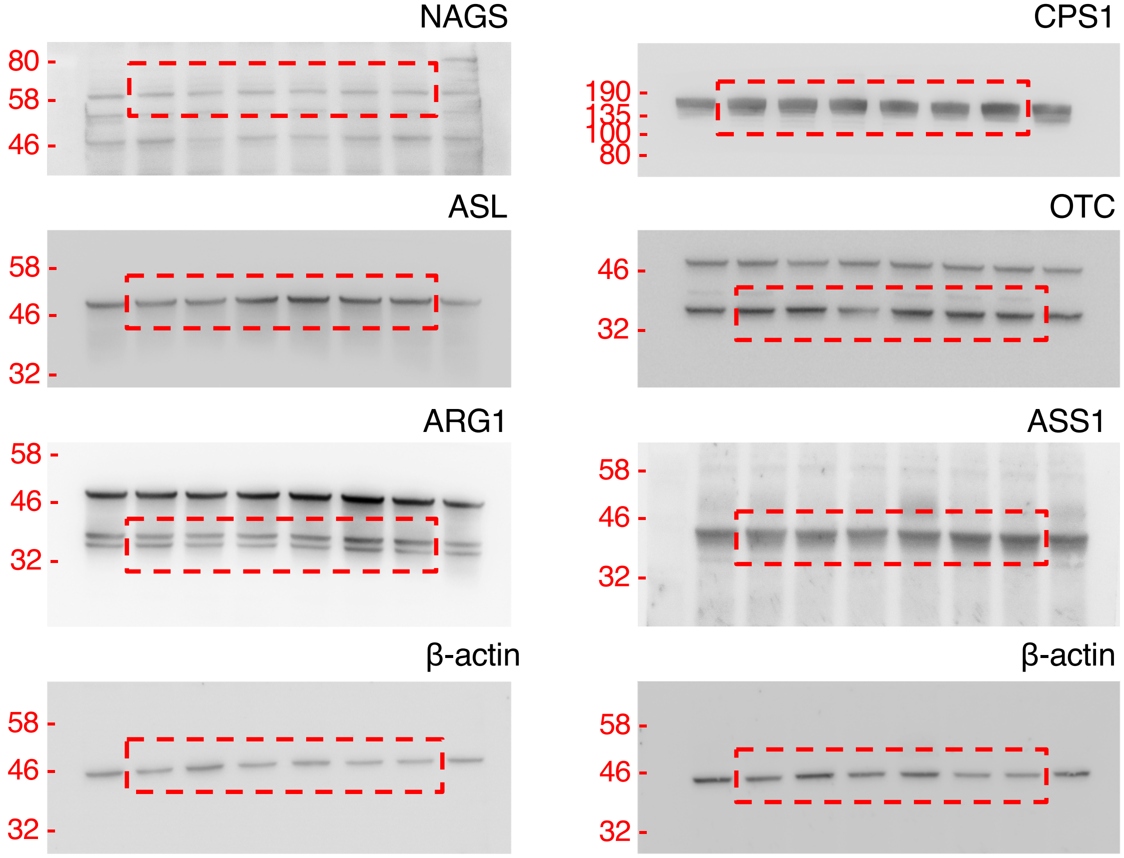

EV. 1C

	NAGS/Actin	CPS1/Actin	OTC/Actin	ASS1/Actin	ASL/Actin	ARG1/Actin
WT	0.31690141	0.78888889	1.433333333	0.6	0.450704225	0.753521127
WT	0.39622642	1.234375	2.203125	0.734375	0.509433962	0.830188679
WT	0.22535211	0.84146341	1.695121951	0.63414634	0.373239437	0.711267606
WT	0.3655914	1.45614035	1.596491228	0.9122807	0.806451613	0.870967742
Becn1F121A	0.25925926	1.29230769	2.046153846	0.76923077	0.731481481	0.833333333
Becn1F121A	0.40740741	1.16666667	1.939393939	0.86363636	0.851851852	1.296296296
Becn1F121A	0.44871795	1.55172414	2.224137931	1.05172414	0.794871795	1.346153846
Becn1F121A	0.37113402	1.17808219	1.684931507	0.80821918	0.494845361	0.865979381
