# Supplementary material for: Beclin‐1‐mediated activation of autophagy improves proximal and distal urea cycle disorders
Source: EMBO Mol Med. 2020 Dec 28;13(2):e13158. doi: 10.15252/emmm.202013158 (PMC7863400; doi:10.15252/emmm.202013158)
Supplement: Supplementary file 3 — Source Data for Expanded View [file EMMM-13-e13158-s007.zip › SD_EV3.pdf]

EV. 3A

Age (days)	WT + Vehicle									Body weight (g)					
12	9.5	7.58	7.39	5.98	6.49	6.39	7.4	7.82							
13	9.79	7.77	7.68	6.37	6.89	6.87	8.22	8.4							
14	10.2	8.12	8.07	6.79	7.35	7.23	8.97	9.06							
15	10.35	8.33	8.39	7.03	7.63	7.58	9.72	9.96							
16	10.75	9.07	8.97	7.24	7.89	7.76	10.1	10.69							
Age (days)	WT+TB-1														
12	12.09	8.65	10.7	6.1	6.38	6.7	4.91	7.81							
13	11.74	9.06	11.1	6.25	6.64	7.21	5.37	7.99							
14	11.34	9.64	11.1	6.54	7.08	7.78	5.67	8.75							
15	11.09	10.1	10.8	6.98	7.48	8.52	6.34	9.6							
16	11.03	10.3	10.6	7.24	7.75	9.06	6.89	10.03							
Age (days)	AslNeo/Neo + Vehicle														
12	4.96	7.58	6.37	7.03	5.13	4.79	6.69	4.64							
13	5.3	7.9	6.99	7.05	5.11	5.22	6.97	4.58							
14	5.85	7.5	7.48	6.82	5.37	5.55	7.37	4.59							
15	6.08	7.26	6.63	6.9	5.51	6.36	7.77	4.78							
16	6.5	7.65		6.75	5.71	6.7	6.66								
Age (days)	AslNeo/Neo + TB-1														
12	5.05	5.95	6.81	8.75	5.5	5.99	5.48	5.49	5.93	6.07	5.58	5.58	5.51	4.84	6.16
13	5.5	6.41	7.06	8.72	5.76	6.02	6.13	6.12	6.54	6.27	5.88	5.61	5.71	5.07	6.55
14	6.1	6.78	7.69	8.59	6.1	6.25	6.51	6.19	7.21	6.87	5.7	5.83	6.05	5.34	6.67
15		7.3	6.26	8.33	6.35	6.75	7.11	6.76	6.51	7.43	5.99	6	5.98	5.74	6.85
16		7.52		8.51	6.48	7.04	7.46	7.15		7.95	5.81	6.31	6.22	5.78	6.9

EV. 3B

WT (Vehicle + TB-1)	AslNeo/Neo + Vehicle	AslNeo/Neo + TB-1
37	320	94
48	63	69
11	27	48
7	126	7
21		148
25		117
		7
		7
		34
		2
		0
Blood ammonia (µM)		

EV. 3C

WT + Vehicle	WT + TB-1	AslNeo/Neo + Vehicle	AslNeo/Neo + TB-1
0.303751857	0.411877676	0.084442114	0.056482307
0.504931411	0.263778069	0.105193534	0.093834862
0.741480122	0.564415902	0.184599388	0.19316208
0.435468764	0.61640367	0.088592398	0.198972477
		0.069588467	0.220990826
			0.232917431
			0.106285714
	Fumarate (nmol/µg)/mg protein		
