# Supplementary material for: Beclin‐1‐mediated activation of autophagy improves proximal and distal urea cycle disorders
Source: EMBO Mol Med. 2020 Dec 28;13(2):e13158. doi: 10.15252/emmm.202013158 (PMC7863400; doi:10.15252/emmm.202013158)
Supplement: Supplementary file 3 — Source Data for Expanded View [file EMMM-13-e13158-s007.zip › SD_EV2.pdf]

EV. 2A

spf-ash + Vehicle Day 0	spf-ash + TB-1 Day 0	spf-ash + Vehicle Day 10	spf-ash + TB-1 Day 10
27.7	23.5	27	22.9
27.5	26	26.5	24.4
26	25.5	23	24.8
23	25	23	25.9
24	26	23	29.3
Body weight (g)			

EV. 2B

WT	spf-ash + Vehicle	spf-ash + TB-1
45.8	1.6	2
49.6	1.3	1.9
45.5	1.4	1.3
	1.2	1.3
	0.9	0.7
	µM citrulline/µg protein/30 min	

EV. 2C

days	spf-ash untreated					spf-ash + TB-1					spf-ash + NaBz + l-arg +TB-1						spf-ash + NaBz + l-arg					WT			
0	23	21	17	23	29	26	24	24.5	25.7	23	24	23	20	21.2	19	19	18	14	18	18	19	32	33	38	32
1	23	21	17	23	29	26	24	24.5	25.7	23	24	23	19	21.2	19	19	16	14	18	18	19	32	33	38	32
2	22	19	16	21	29	25	22.1	22.7	23.4	20	21.1	21	17	18.6	18	18	15	12	16.5	14	16	30.8	33	38.2	31.3
3	21	17.9	15	19.5	27	23.3	21.1	21	22.2	18.3	20	21	18	16.8	17	17	13	11.4	15.4	12	14	30	32.7	37.5	31
4	20	13.5	14	18	25.3	21.5	19.1	20	21.1	17	19	20	17	17	16	15	12	10	14.5	11.6	13	30	33	36	30
5	18.8	12.6	13	16.6	24	20	17	19	20.2	15	18	19	16	15.8	15	14	11.3	9.6	14	10.9	13	31	33	37	32.8
6	16.6	14.5		15	22	17.8	16	17.8	18.9		17	18	15	15	14	13		8.8	13.5		12	30	32	36.8	31
7	15.6				20	17.4	15.3	16.9	18.1		15	15	15	13.5	14	13			12			30.8	32.1	36.2	30.3
8					17.3			15.7	16.7		15	15	14	12.8	13	12			11.8			30.6	32	37	30
9									16		14.6		14	11.8	13				11.2			32	30	34	29.5
10											14.5		14		12							29.7	31.1	34	29.5

Body weight (g)

EV. 2D

WT D0	spf-ash untreated D0	spf-ash + TB-1 D0	spf-ash + NaBz + l-Arg D0	spf-ash + NaBz + l-Arg + TB-1 D0
39	897.6	420.3	779.944	612.3414
74.6	674.1	414.2	202.945	396.6985
94.6	284.7	1720.2	161.8142	644.2286
128.1	333.33	796.9	413.377	514.83
	674.08	1681.6	292.4842	818.2624
				559.8468
WT D4	spf-ash untreated D4	spf-ash + TB-1 D4	spf-ash + NaBz + l-Arg D4	spf-ash + NaBz + l-Arg + TB-1 D4
42.4	2534	700.73	7270.812	366.7522
34.1	659.03	799.91	4087.002	73.71634
102.3	2231.46	436.72	1503.143	2417.583
78.1	1004.6	1310.38	1313.683	1137.275
	3160.4	1340.91	2325.64	1087.241
				265.3391
µM Orotic acid/mM creatinine				
