# Supplementary material for: Beclin‐1‐mediated activation of autophagy improves proximal and distal urea cycle disorders
Source: EMBO Mol Med. 2020 Dec 28;13(2):e13158. doi: 10.15252/emmm.202013158 (PMC7863400; doi:10.15252/emmm.202013158)
Supplement: Supplementary file 5 — Source Data for Figure 1 [file EMMM-13-e13158-s003.pdf]

Fig. 1A

WT Baseline	Becn1F121A Baseline	WT 30 min Pi	Becn1F121A 30 min Pi
290.1364116	272.6611579	1843.270023	834.3469446
233.1842885	345.5000725	2427.374248	989.5711101
282.9830569	359.4365321	2593.613603	1683.459179
374.0116004	322.1509054	3129.756744	2401.74538
306.1454198	236.1017711	2999.931104	2313.439173
203.6585399	262.3500874	2377.098823	2138.264439
142.8319186	230.235789	3122.473762	2057.952815
283.7520388	353.8589913	2549.160065	1968.270843
Blood ammonia (µM)			

Fig. 1B

spf-ash + Vehicle					spf-ash + TB-1				
Day 0	Day 1	Day 4	Day 8	Day 10	Day 0	Day 1	Day 4	Day 8	Day 10
700.9601	377.2591	771.5748	89.67558	299.5416	679.8077	141.1861	299.4774	142.0651	153.2934
258.617	130.2333	522.3829	148.824	330.1138	318.4513	42.4337	233.4595	182.8107	355.9944
372.4966	503.7032	277.6981	274.1458	327.7887	365.9144	65.01695	356.7746	59.34322	197.9089
128.6939	444.2434	206.2448	511.7356	456.5062	403.0979	80.79408	397.6998	47.69653	196.1395
587.9885		541.4418	497.0891	586.9855	515.0648	100.8085	424.9367	334.1315	205.7872
	µM Orotic acid/mM creatinine								

Fig. 1C

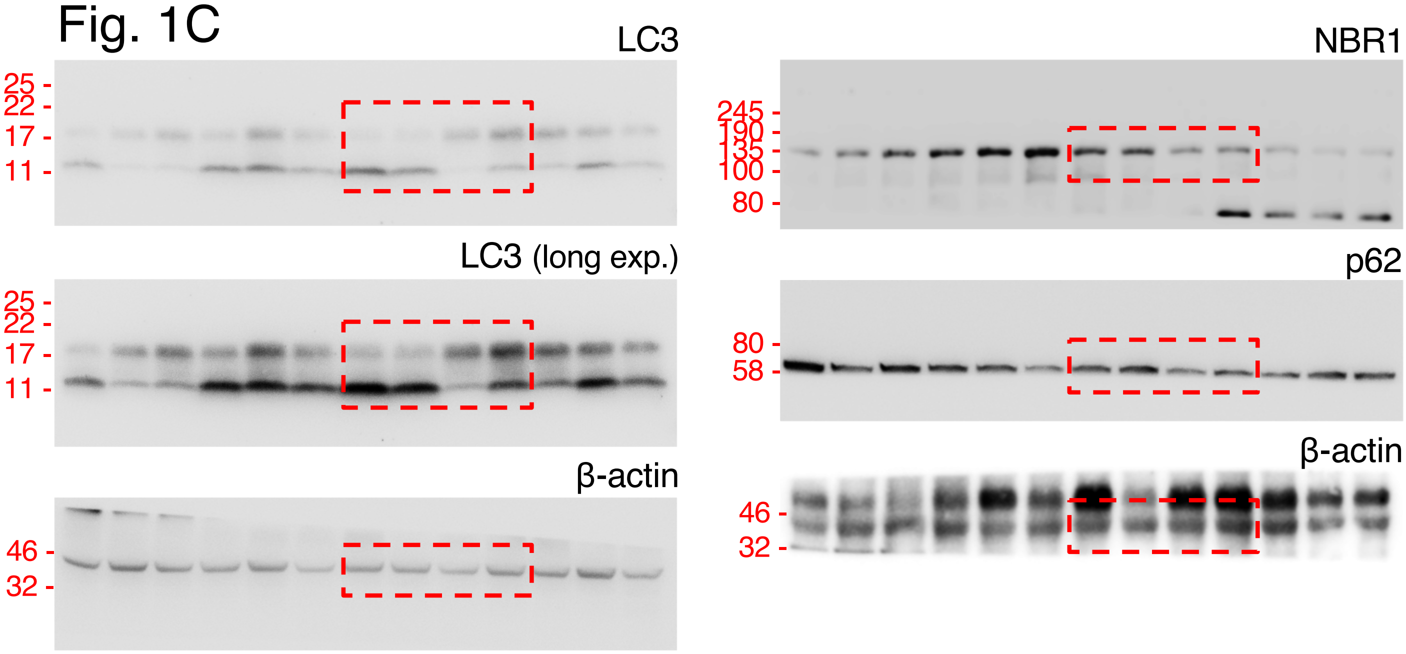

Fig. 1D

LC3II/Actin	
spf-ash + Vehicle	spf-ash + TB-1
1	0.45454545
1.01030928	0.54081633
1.18965517	0.42
1.22988506	0.66141732
0.98780488	0.48888889
NBR1/Actin	
spf-ash + Vehicle	spf-ash + TB-1
0.496350365	0.32
0.482758621	0.325581395
0.407142857	0.235714286
0.365079365	0.225
0.350364964	0.220125786
p62/Actin	
spf-ash + Vehicle	spf-ash + TB-1
0.702380952	0.560606061
0.577319588	0.551020408
0.74137931	0.47
0.551724138	0.283464567
0.597560976	0.411111111

Fig. 1F

WT	spf-ash untreated	spf-ash + TB-1	spf-ash + NaBz + l-Arg	spf-ash + NaBz + l-Arg + TB-1
233.44	431.51	481.03	525.2	0
159.16	360.77	537.62	8.8	26.5
201.61	650.8	304.18	14.1	88.4
217.52	459.81	226.37	15.9	28.3
	594.21	339.55	141.5	0
				0
Blood ammonia (µM)				
