# Supplementary material for: Beclin‐1‐mediated activation of autophagy improves proximal and distal urea cycle disorders
Source: EMBO Mol Med. 2020 Dec 28;13(2):e13158. doi: 10.15252/emmm.202013158 (PMC7863400; doi:10.15252/emmm.202013158)
Supplement: Supplementary file 6 — Source Data for Figure 2 [file EMMM-13-e13158-s004.pdf]

Fig. 2B

AslNeo/Neo + Vehicle	AslNeo/Neo + TB-1
196	454
125	149
237	153
38	178
53	226
48	237
84	293
114	240
47	111
	328
	219
	53
	55
	114
	132
^{15}N-Urea (µM)	

Fig. 2C

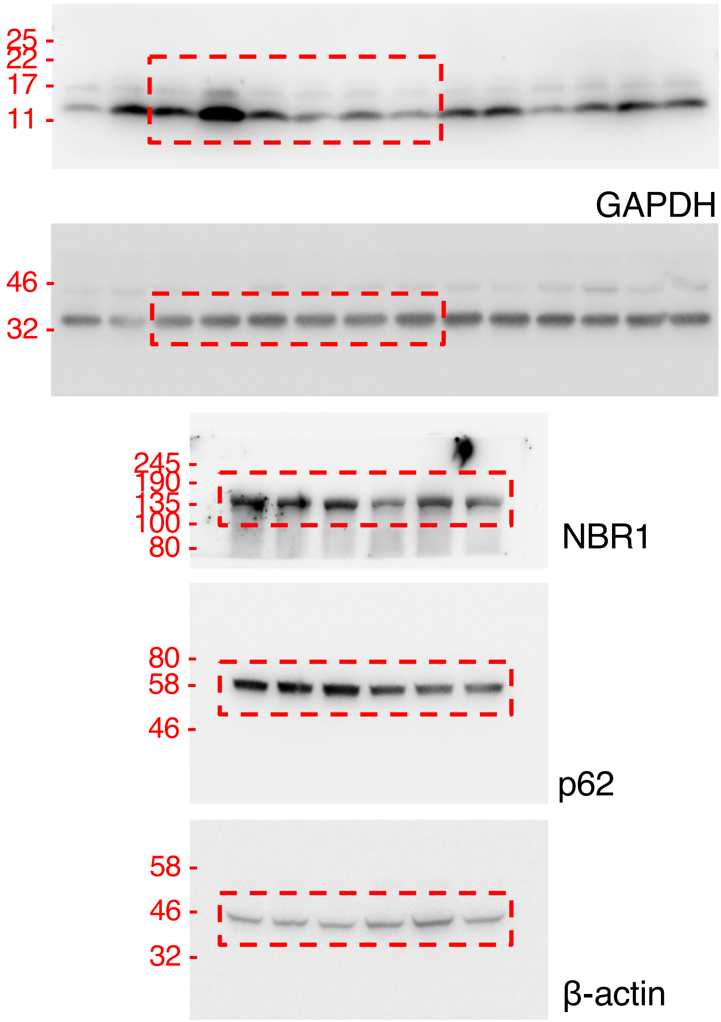

Fig. 2D

LC3II/GAPDH	
AslNeo/Neo + Vehicle	AslNeo/Neo + TB-1
0.590361446	0.891891892
2.440677966	1.01369863
1.726027397	0.421686747
2.511111111	1.164705882
1.561797753	1.16091954
	0.67032967
	0.87654321
	1.065934066
	0.948979592
NBR1/Actin	
AslNeo/Neo + Vehicle	AslNeo/Neo + TB-1
1.68	1.112676056
1.604938272	1.063291139
1.581081081	1.1625
1.636363636	1.123595506
0.731182796	1.242857143
	0.623853211
	0.519230769
	1.221052632
	0.571428571
p62/Actin	
AslNeo/Neo + Vehicle	AslNeo/Neo + TB-1
1.573333333	2.436619718
2.456790123	2.240506329
2.72972973	1.425
5.272727273	1.438202247
1	1.028571429
	0.532110092
	0.384615385
	0.684210526
	0.242857143

Fig. 2E

WT + Vehicle	WT + TB-1	AslNeo/Neo + Vehicle	AslNeo/Neo + TB-1
48	51	464	412
38	31	651	458
39	29	1084	386
48	42	642	713
38			927
			462
			268
			313
			277
			354
			45
			549
			102
			58
	Argininosuccinate in DBS (µM)		

Fig. 2F

AslNeo/Neo + Vehicle	AslNeo/Neo + TB-1
11.30438675	8.949865712
13.25604297	10.97314235
25.23455685	4.518352731
53.7484333	9.863025962
10.24798568	15.20769919
	23.77529096
	9.36168308
	0.445554
	13.87568
	3.376588
	3.467332
	Liver content of 15N-Argininosuccinate (µM)
