# Supplementary material for: Beclin‐1‐mediated activation of autophagy improves proximal and distal urea cycle disorders
Source: EMBO Mol Med. 2020 Dec 28;13(2):e13158. doi: 10.15252/emmm.202013158 (PMC7863400; doi:10.15252/emmm.202013158)
Supplement: Supplementary file 7 — Source Data for Figure 3 [file EMMM-13-e13158-s005.pdf]

Fig. 3A

Liver H&E staining

Scale bar 100 µm

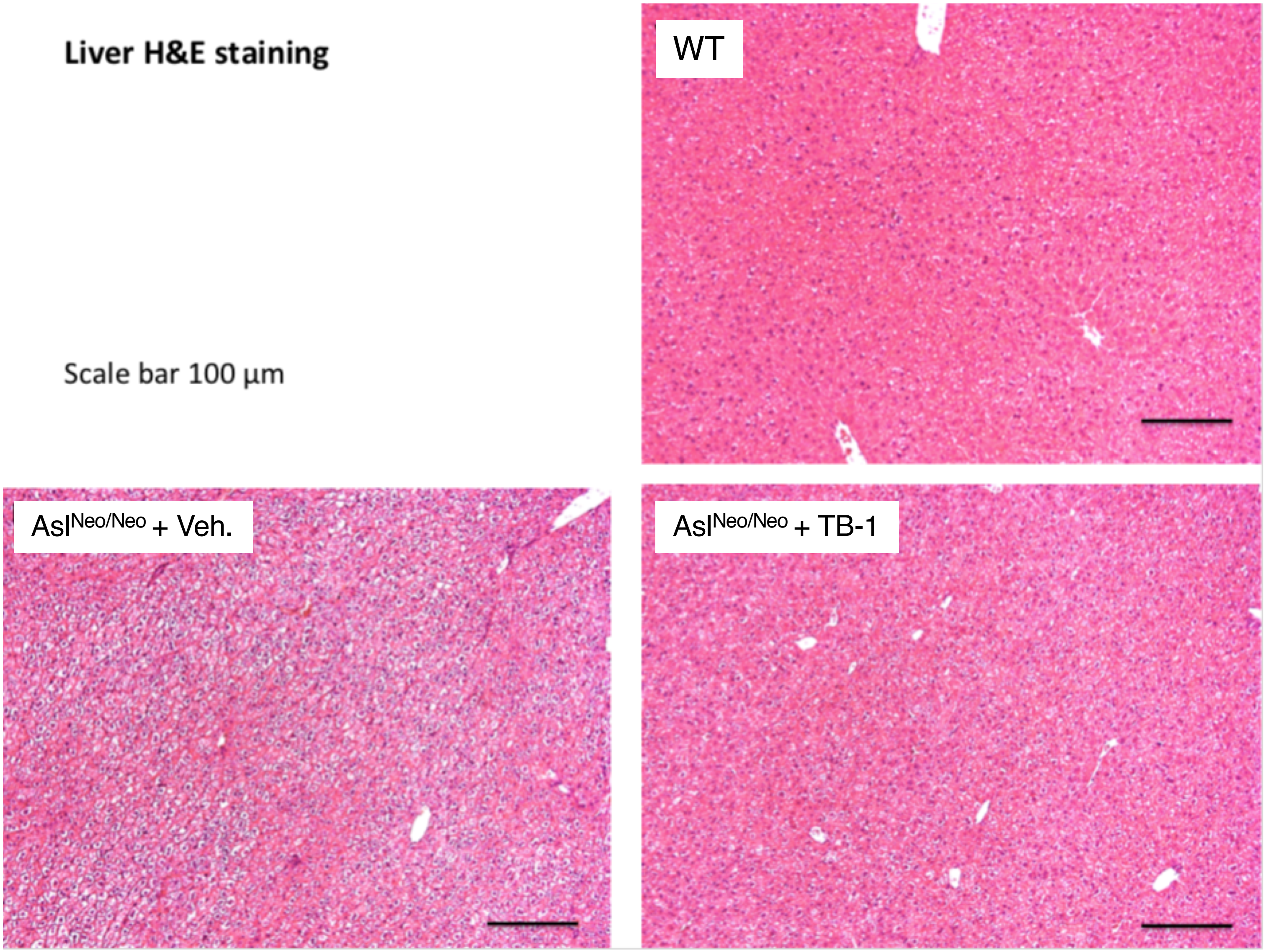

Fig. 3A (cont.)

Liver glycogen storage

PAS staining

Scale bar 100 µm

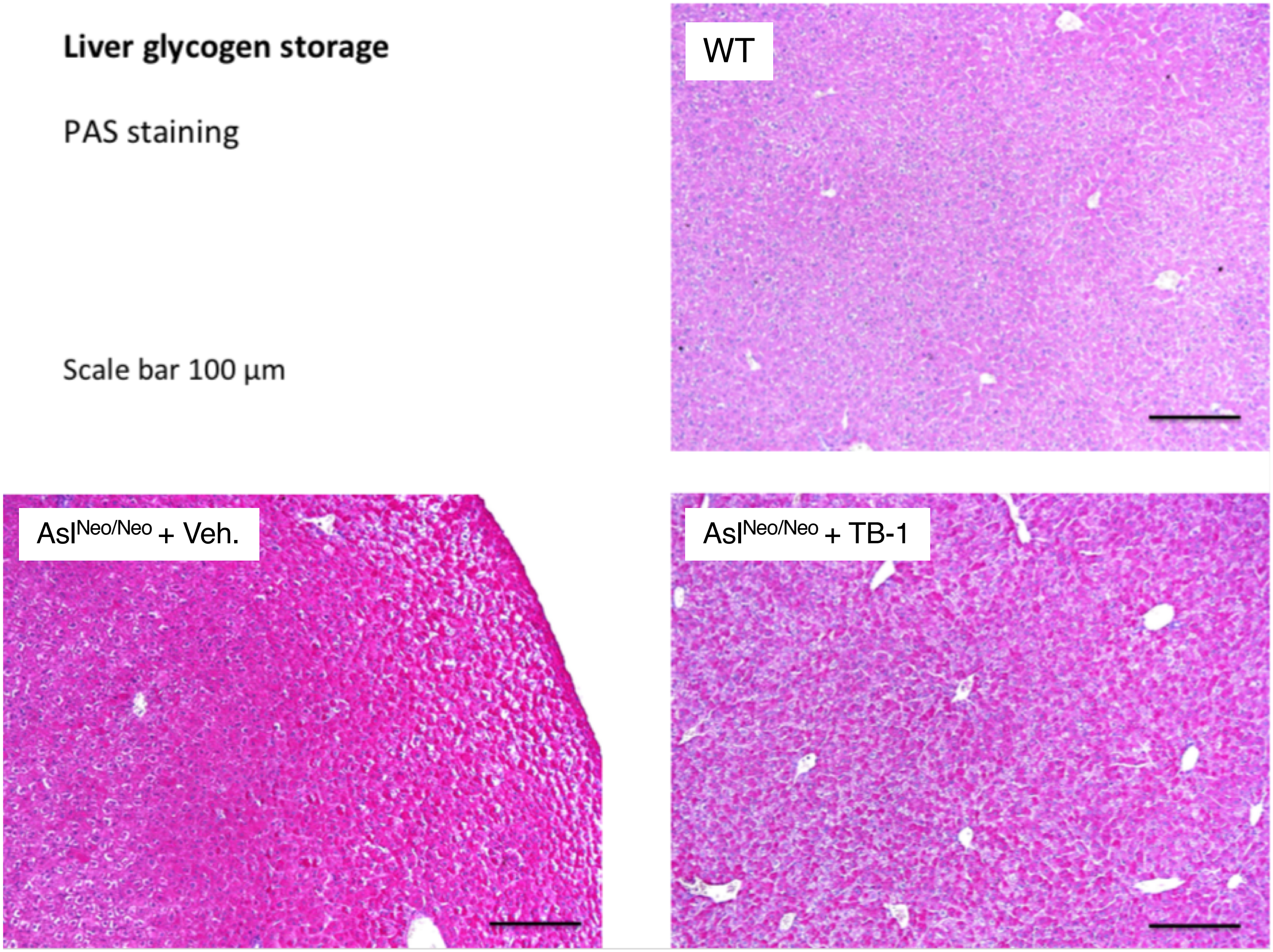

Fig. 3B

WT + Vehicle	AslNeo/Neo + Vehicle	AslNeo/Neo + TB-1
7.46	51.66	49.64
2.96	73.68	22.98
5.08	69.73	41.29
7.36	49.8	25.37
17.63		
	Glycogen (% immunoreactivity/mm^2)	

Fig. 3C

WT + Vehicle	AslNeo/Neo + Vehicle	AslNeo/Neo + TB-1
1168.086271	4284.387589	1245.808865
359.446216	7969.354218	2999.921843
771.5651213	2957.290068	4225.169293
310.2347243	7318.14338	5206.052646
2744.073694	6740.2259	4166.746754
	5500.616187	4787.356337
		4184.060564
		3985.647797
		6523.759806
		4007.880121
		520.754898
		2509.984414
	Glycogen (µg/mg protein)	

Fig. 3D

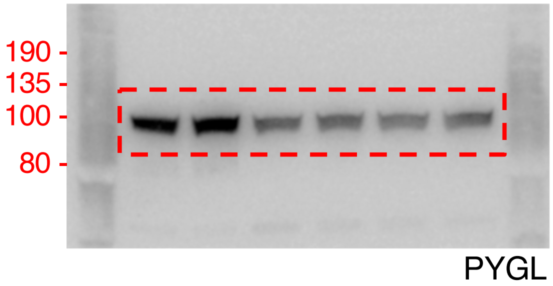

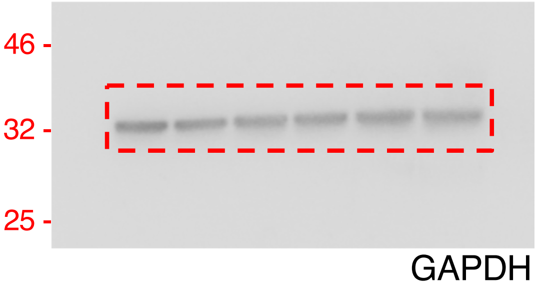

PYGL/GAPDH		
WT + Vehicle	AslNeo/Neo + Vehicle	AslNeo/Neo + TB-1
2.058823529	1.607142857	1.0625
4.473684211	1.357142857	1.620689655
2.777777778	1.073170732	0.474576271
1.965517241	0.673076923	0.196969697
