# Supplementary material for: Beclin‐1‐mediated activation of autophagy improves proximal and distal urea cycle disorders
Source: EMBO Mol Med. 2020 Dec 28;13(2):e13158. doi: 10.15252/emmm.202013158 (PMC7863400; doi:10.15252/emmm.202013158)
Supplement: Supplementary file 8 — Source Data for Figure 4 [file EMMM-13-e13158-s006.pdf]

Fig. 4B

ASLNeo/Neo + vehicle				ASLNeo/Neo + TB-1				WT + Vehicle		
total nuclei (number)	nuclei (w/o glycogen)	nuclei (with glycogen)		total nuclei (number)	nuclei (w/o glycogen)	nuclei (with glycogen)		total nuclei (number)	nuclei (w/o glycogen)	nuclei (with glycogen)
56	27	29		43	33	10		53	53	0
100%	48%	52%		100%	76.70%	23.30%		100%	100%	0%

ASLNeo/Neo + vehicle				ASLNeo/Neo + TB-1				WT + Vehicle		
total nuclei (number)	nuclei (w/o glycogen)	nuclei (with glycogen)		total nuclei (number)	nuclei (w/o glycogen)	nuclei (with glycogen)		total nuclei (number)	nuclei (w/o glycogen)	nuclei (with glycogen)
67	29	38		58	38	20		48	43	5
100%	43.00%	56.70%		100%	65.50%	34.50%		100%	90%	10%

ASLNeo/Neo + vehicle				ASLNeo/Neo + TB-1				WT + Vehicle		
total nuclei (number)	nuclei (w/o glycogen)	nuclei (with glycogen)		total nuclei (number)	nuclei (w/o glycogen)	nuclei (with glycogen)		total nuclei (number)	nuclei (w/o glycogen)	nuclei (with glycogen)
69	51	18		72	51	21		67	56	11
100%	73.30%	26.70%		100%	70.80%	29.20%		100%	83.60%	16.40%
